# Pre-exposure Prophylaxis Persistence at a Diverse Sexual Health Clinic: Comparison of the pre-COVID-19 era to the COVID-19 era

**DOI:** 10.1007/s10461-023-03996-3

**Published:** 2023-02-04

**Authors:** Laura Platt, Fatma M. Shebl, Yiqi Qian, Nicholas Spanos, Cody P. Nolan, Kevin L. Ard, Ingrid V. Bassett

**Affiliations:** 1 Division of Infectious Diseases, Brigham and Women’s Hospital, Boston, MA USA; 2 Medical Practice Evaluation Center, Massachusetts General Hospital, Boston, MA USA; 3 Department of Internal Medicine, Brigham and Women’s Hospital, Boston, MA USA; 4 Division of Infectious Diseases, Massachusetts General Hospital, Boston, MA USA

**Keywords:** PrEP, COVID-19, Disparities, Telehealth

## Abstract

The COVID-19 pandemic interrupted health care delivery and exacerbated disparities. Many sexual health clinics transitioned to telemedicine, including for pre-exposure prophylaxis (PrEP). We conducted a retrospective cohort study of patients at an urban sexual health clinic to assess the likelihood and predictors of PrEP persistence in the year following PrEP initiation. We compared patients starting PrEP in the four months preceding the first COVID surge to those starting PrEP one year prior. We found lower PrEP persistence in the COVID cohort compared to the pre-COVID cohort (50.8% vs. 68.9%, respectively). In both cohorts, most care was provided through in-person visits and telemedicine was rare. In the pre-COVID cohort, older patients and those identifying as non-Hispanic White were more likely to persist on PrEP. In the COVID cohort, these disparities in PrEP persistence were not observed. Flexible models of care may facilitate equitable care engagement and re-engagement.

## Introduction

The COVID-19 pandemic interrupted many aspects of health care and exacerbated existing racial and ethnic disparities [1–3]. Among health care services, sexual health care was significantly impacted, with many clinics unable to continue offering in-person visits for the testing and treatment of sexually transmitted infections (STIs) or the provision of pre-exposure prophylaxis for HIV (PrEP) [4–7]. Available data suggest that in the early months of the COVID-19 pandemic, patients experienced lapses in PrEP refills and decreased PrEP initiations [8–10]. On May 15, 2020, the CDC issued a Dear Colleague letter acknowledging the need for ongoing HIV prevention services and the challenges of providing these services while minimizing non-urgent health care encounters [11]. In this letter, the CDC recommended transitioning to telehealth services for PrEP provision (tele-PrEP) with lab-only visits or at-home testing options. Many clinics transitioned to telemedicine and some have described their early experiences in the pandemic [7–9, 12, 13]. Some clinics found that the transition to tele-PrEP was better at preserving PrEP access for patients who are older and identify as non-Hispanic White [8].

Unlike many clinics in our region that transitioned to telehealth services at the onset of the COVID pandemic, our clinic remained open for in-person visits and offered optional telehealth services as an alternative method of care. Data are sparse regarding characteristics of PrEP persistence within a diverse clinic population that maintained access to in-person care. It is not known whether adherence to PrEP standards of care (routine safety labs and STI and HIV screening) changed during the pandemic, and it is not known whether PrEP persistence during the pandemic is similarly associated with age, race, and ethnicity within a largely in-person clinic setting.

In this study, we conducted a single-center retrospective cohort study to compare the likelihood of PrEP persistence between patients newly started on PrEP just before the onset of the pandemic (COVID-cohort) with that of patients initiated on PrEP one year prior (pre-COVID cohort). Among PrEP persisters, the likelihood of adherence to safety labs and STI screening was compared. We compared differences in sociodemographic characteristics among PrEP persisters and non-persisters. Finally, we used multivariable robust Poisson regression to identify predictors of PrEP persistence.

## Methods

We conducted a retrospective review of patients at a sexual health clinic in Boston, Massachusetts, to assess PrEP persistence and characteristics of care for a cohort of patients initiating PrEP for the first time in the four months prior to the initial surge of COVID-19 in our city, which began in early March 2020 (COVID cohort, November 1, 2019 – February 29, 2020). This cohort was compared to a pre-COVID cohort of patients who initiated PrEP for the first time one year prior (pre-COVID cohort, November 1, 2018 – February 28, 2019). We used manual chart reviews to ascertain outcomes for each patient for one year from the date of PrEP initiation.

Our clinic, the Massachusetts General Hospital Sexual Health Clinic, is a safety net clinic with locations on an academic medical campus in Boston, Massachusetts and at a health center in Chelsea, Massachusetts. Our clinic provides comprehensive sexual health testing, treatment, and prevention services at no cost to patients. Our clinic sees an average of 338 patients per month, and at approximately half of all visits, patients have a CDC indication for PrEP. Data are systematically collected at each clinic visit, including patient demographics (age, sex assigned at birth, gender identity, race, ethnicity, country of birth, address) and risk-associated behaviors (substance use, sexual practices). Our study included all patients in our clinic who were prescribed PrEP for the first time at our clinic during the time periods outlined above. This included patients who started PrEP after a course of post-exposure prophylaxis (PEP) as well as those who had previously been prescribed PrEP by other providers. As part of routine care, all patients were confirmed to be HIV negative before starting PrEP, and only medications that were FDA-approved for PrEP at the time of our study (tenofovir disoproxil fumarate and emtricitabine or tenofovir alafenamide and emtricitabine) were used. Our primary outcome of interest was PrEP persistence, defined as any prescription for PrEP provided more than 60 days after the initial prescription. We subclassified PrEP persistence into three categories: continuous (at least one 90-day prescription every 120 days), short-term (a single prescription within 120 days of initiation), and interrupted (any gap of greater than 120 days for those with at least two 90-day prescriptions). This definition of persistence aligns with other studies that define medication lapses as greater than 30 days beyond a medication ‘due date,’ and in our population would equate to medication availability of at least five doses per week, which is a dosing frequency found to be highly effective against HIV acquisition among MSM [14, 15]. Patients who reported transferring PrEP care to another clinic were considered PrEP persisters but were not subclassified. When patients reported discontinuing PrEP (either for the duration of the study period or temporarily), reasons for discontinuation were recorded.

We compared the cumulative incidence of PrEP persistence between pre-COVID and COVID cohorts. For PrEP persisters, we also compared characteristics of follow-up care between the two cohorts, including adherence to recommended screening (HIV and bacterial STI testing with each prescription, serum creatinine testing every six months) and use of telehealth services (video visits, telephone visits, and routine telephone contact). Lastly, we compared demographic and behavioral predictors of PrEP persistence in each cohort, including age, race/ethnicity, sex assigned at birth, place of birth (US-born or non-US-born), Neighborhood Deprivation Index (NDI) as a proxy for neighborhood-level socioeconomic status, injection and non-injection drug use, transactional sex, number of sexual partners, and diagnosis of bacterial STIs.

### Statistical Methods

We compared baseline characteristics and primary outcomes between cohorts. Additionally, within each cohort, we compared demographics and behaviors among people who did and did not persist on PrEP. These comparisons were performed using Fisher exact tests and Kruskal-Wallis for categorical and continuous variables, respectively. We employed multivariable robust Poisson regression to estimate the adjusted cumulative incidence ratios (aIRs) and the 95% confidence intervals (95% CIs) for PrEP persistence. To select the final multivariable Poisson models, we used four steps. (1) We assessed an *a priori* list of effect modifiers (age, race/ethnicity, sex assigned at birth, place of birth, NDI, bacterial STI in the past year, and condom use during the follow-up period) based on clinical importance and review of the literature. We used robust Poisson regression to assess the interaction between potential effect modifiers and PrEP persistence. A variable was deemed an effect modifier if the p-value of the interaction term was < 0.05. (2) We assessed an *a priori* list of confounders (age, sex assigned at birth, place of birth, NDI, bacterial STI in the past year, injection and non-injection drug use, transactional sex, and number of sex partners) using the change of the beta approach in which we calculated the difference between crude and adjusted regression coefficient betas divided by the adjusted regression coefficient beta. If the relative change was greater than 10%, we considered the variable to be a confounder. (3) We included the variables identified from steps 1 and 2 and variables that are frequently used as clinical predictors in a saturated model, and (4) we fitted several reduced models that included at least the confounders identified in step 2, and identified the model that best fits the data using the Quasi-likelihood under the Independence model Criterion (QIC) statistic, which is analogous to the Akaike information criterion (AIC) [16]. Lower QIC indicates better model fit. We followed the suggestion of Rothman and others and did not adjust for multiple comparisons [17, 18].

We calculated a Neighborhood Deprivation Index (NDI) as a proxy for neighborhood-level socioeconomic status. This process has been described more fully by Messer et al. [19] and has been previously used to characterize patients on PrEP [20]. Briefly, based on zip codes of patient home addresses, we extracted data from the American Community Survey from the U.S. Census [21]. We used twenty-five variables in total, including twenty variables used by Messer et al. [19] and five variables that we identified (median household income, population density, average household size, percent of nonessential workers, and percent insured). We used principal component analysis to calculate NDI as previously described. The NDI variable has a mean of zero and a standard deviation of one, with lower values of NDI indicating a lower socioeconomic status (“more deprived”) neighborhood. All analyses were conducted using SAS 9.4 software (Copyright, SAS Institute Inc. Cary, NC, USA).

This study was approved by the Partners Human Subjects Research Committee (Protocol 2018P001340, Boston, MA).

## Results

There were 74 patients in the pre-COVID cohort and 63 patients in the COVID cohort. Baseline demographic and behavioral characteristics of these cohorts are shown in Table 1. Both cohorts had a median age of 28 years. Most patients identified as male (91%) and reported having sex with men (88%). Approximately half of the patients were non-Hispanic White (47%), and approximately half were foreign-born (49%). In the year prior to PrEP initiation, a larger proportion of the pre-COVID cohort had been diagnosed with a bacterial STI compared to the COVID cohort (97% and 83%, respectively), and a larger proportion of the pre-COVID cohort engaged in transactional sex compared to the COVID cohort (8% and 0%, respectively). Although not statistically significant, a larger proportion of the pre-COVID cohort had more than ten sexual partners reported in the year prior to PrEP initiation compared to the COVID cohort (60% and 48%, respectively).

**Table 1:** Baseline patient characteristics by cohort

	Total	Pre-COVID cohort	COVID cohort	Test statistic	P-value
	(N = 137)	(N = 74)	(N = 63)		
Age in years, median (IQR)	28 (24–33)	28 (24–36)	28 (24–33)	0.282^&^	0.595
Gender identity, n (%)				0.014^&&^	0.636
Male	124 (91)	67 (91)	57 (91)		
Female	7 (5)	4 (5)	3 (5)		
Trans male	1 (< 1)	1 (1)	0 (0)		
Trans female	0 (0)	0 (0)	0 (0)		
Nonbinary	3 (2)	2 (3)	1 (2)		
Other	2 (2)	0 (0)	2 (3)		
Race/ethnicity, n (%)				4.427^&^	0.351
Non-Hispanic White	64 (47)	38 (51)	26 (41)		
Non-Hispanic Black	14 (10)	5 (7)	9 (14)		
Hispanic/Latinx	40 (29)	20 (27)	20 (32)		
Other	18 (13)	11 (15)	7 (11)		
Missing	1 (< 1)	0 (0)	1 (2)		
Place of birth, n (%)				0.026^&&^	0.351
US-born	68 (50)	36 (49)	32 (51)		
Foreign-born	67 (49)	38 (51)	29 (46)		
Missing	2 (2)	0 (0)	2 (3)		
Neighborhood Deprivation Index, median (IQR)	-0.1 (-1–0.8)	-0.1 (-1–0.8)	0.2 (-0.5–0.7)	0.030^&^	0.863
PrEP risk group, n (%)				0.036^&&^	0.829
MSM	120 (88)	64 (87)	56 (89)		
IDU	0 (0)	0 (0)	0 (0)		
MSM/IDU	4 (3)	3 (4)	1 (2)		
Heterosexual	1 (< 1)	1 (1)	0 (0)		
Other/missing	12 (9)	6 (8)	6 (10)		
Bacterial STI diagnosed in the previous year, n (%)				8.629^&^	0.003
Yes	124 (91)	72 (97)	52 (83)		
No	13 (10)	2 (3)	11 (18)		
Number of sexual partners in the previous year, n (%)				0.006^&&^	0.395
1–5	31 (23)	15 (20)	16 (25)		
6–10	31 (23)	15 (20)	16 (25)		
> 10	74 (54)	44 (60)	30 (48)		
Missing	1 (< 1)	0 (0)	1 (2)		
Non-injection drug use in the previous year, n (%)				3.778^&^	0.052
Yes	31 (23)	12 (16)	19 (30)		
No	106 (77)	62 (84)	44 (70)		
Transactional sex in the previous year, n (%)				0.023^&&^	0.031
Yes	6 (4)	6 (8)	0 (0)		
No	131 (96)	68 (92)	63 (100)		

Notes: ^&^ Chi-square value from chi-square test.

^&&^ Table probability value from Fisher exact test.

Frequencies of PrEP persistence are shown in Table 2. A larger proportion of patients in the pre-COVID cohort persisted on PrEP compared to the COVID cohort (69% and 51%, respectively). Among those who persisted on PrEP in the pre-COVID cohort, most had interrupted PrEP (21/51, 41%) or continuous PrEP (17/51, 33%). In contrast, among those who persisted on PrEP in the COVID cohort, most had interrupted PrEP (18/32, 56%) and very few had continuous PrEP (8/32, 25%). In both cohorts, the smallest proportions of patients had short-term PrEP or transferred care. Most patients did not provide a reason for PrEP discontinuation, but among those who did, changed behavior was more common in the COVID cohort than the pre-COVID cohort (13% and 5%, respectively).

**Table 2:** Characteristics of PrEP persistence by cohort

	Total	Pre-COVID cohort	COVID cohort	Test statistic	P-value
	(N = 137)	(N = 74)	(N = 63)		
Persistence status, n (%)				< 0.001^&&^	0.141
Non-persistence	54 (39)	23 (31)	31 (49)		
Any persistence	83 (61)	51 (69)	32 (51)		
Short term	10 (7)	6 (8)	4 (6)		
Interrupted	39 (29)	21 (28)	18 (29)		
Continuous	25 (18)	17 (23)	8 (13)		
Care transferred	9 (7)	7 (10)	2 (3)		
Timing of PrEP lapse^*^, n (%)				5.526^&^	0.137
First six months after PrEP start	73 (53)	33 (45)	40 (64)		
Second six months after PrEP start	29 (21)	17 (23)	12 (19)		
Both first and second six months	10 (7)	7 (10)	3 (5)		
Neither (continuous PrEP)	25 (18)	17 (23)	8 (13)		
Reason for discontinuation, n (%)				9.799^&&^	0.106
Changed behavior	12 (9)	4 (5)	8 (13)		
Poor tolerance	6 (4)	3 (4)	3 (5)		
Financial	2 (2)	2 (3)	0 (0)		
Moved/Access	3 (2)	1 (1)	2 (3)		
Other	2 (2)	0 (0)	2 (3)		

Notes: * PrEP lapse defined as > 120 days between PrEP prescription. The timing of lapse is defined as day 121 since the last PrEP prescription.

^&^ Chi-square value from chi-square test.

^&&^ Table probability value from Fisher exact test.

Demographics and reported sexual behaviors in the year following PrEP initiation stratified by cohort and PrEP persistence are shown in Table 3. In the pre-COVID cohort, patients who were older and identified as non-Hispanic White or Other were more likely to persist on PrEP than their counterparts. In the COVID cohort, age and race/ethnicity were not associated with PrEP persistence. In the COVID cohort, condom use was associated with PrEP persistence whereas in the pre-COVID cohort, condom use was not associated with PrEP persistence, and most participants did not report condom use during the year following PrEP initiation. In both cohorts, over half of PrEP persisters and nearly a third of non-persisters were diagnosed with a bacterial STI in the year following PrEP initiation.

**Table 3:** Demographics and behaviors by cohort and PrEP persistence

	Pre-COVID Cohort					COVID Cohort				
	N	Persisters	Non-persisters	Test statistic	P-value*	N	Persisters	Non-persisters	Test statistic	P-value**
Age in years, median (IQR)	74	28 (25–37)	25 (23–32)	4.643	0.031	63	28 (24–33)	27 (23–33)	0.068	0.794
Gender identity, n (%)				0.084^&&^	0.648				0.046^&&^	0.416
Male	67	47 (70)	20 (29)			57	30 (53)	27 (47)		
Female	4	2 (50)	2 (50)			3	1 (33)	2 (67)		
Trans male	1	1 (100)	0 (0)			0	0 (0)	0 (0)		
Trans female	0	0 (0)	0 (0)			0	0 (0)	0 (0)		
Nonbinary	2	1 (50)	1 (50)			1	1 (100)	0 (0)		
Other	0	0 (0)	0 (0)			2	0 (0)	2 (100)		
Race/ethnicity, n (%)^#^				< 0.001^&&^	0.014				0.002^&&^	0.359
Non-Hispanic White	38	29 (76)	9 (24)			26	10 (38)	16 (62)		
Non-Hispanic Black	5	1 (20)	4 (80)			9	5 (56)	4 (44)		
Hispanic/Latinx	20	11 (55)	9 (45)			20	13 (65)	7 (35)		
Other	11	10 (91)	1 (9)			7	4 (57)	3 (43)		
Missing	0	0 (0)	0 (0)			1	0 (0)	1 (100)		
Place of birth, n (%)				0.828^&^	0.363				0.084^&^	0.803
US-born	36	23 (64)	13 (36)			32	15 (47)	17 (53)		
Foreign-born	38	28 (74)	10 (26)			29	16 (55)	13 (45)		
Missing	0	0 (0)	0 (0)			2	1 (50)	1 (50)		
Neighborhood deprivation index, median (IQR)	74	-0.1 (-1–1.2)	-0.3 (-1–0.7)	2.117^&^	0.146	63	0.3 (-0.4–0.8)	-0.1 (-1–0.7)	1.258^&^	0.262
Condom use in follow-up period, n (%)^#^				0.416^&&^	1.000				21.875^&^	< 0.001
Yes	4	3 (75)	1 (25)			35	27 (77)	8 (23)		
No/Unknown	70	48 (69)	22 (31)			28	5 (18)	23 (82)		
Bacterial STI diagnosed in follow-up period, n (%)				3.233^&^	0.072				3.671^&^	0.055
Yes	34	27 (79)	7 (21)			28	18 (64)	10 (36)		
No	40	24 (60)	16 (40)			35	14 (40)	21 (60)		

Notes: ^#^ Indicates significant P-value for interaction between cohort and the row variable in models predicting PrEP persistence.

* P-value for the association between the row variable and PrEP persistence in the pre-COVID cohort.

** P-value for the association between the row variable and PrEP persistence in the COVID cohort.

^&^ Chi-square value from chi-square test.

^&&^ Table probability value from Fisher exact test.

Characteristics of care for patients persisting on PrEP are shown in Table 4. Recommended screening was performed for a majority of patients in both cohorts, although slightly more in the pre-COVID cohort than the COVID cohort; recommended HIV and STI testing occurred in 88% and 75% respectively, and serum creatinine was measured in 77% and 69% respectively. For both cohorts, the majority of follow-up visits were in-person (79%), although the overall percentage was smaller in the COVID cohort compared to pre-COVID (68% and 85%, respectively). All other telehealth methods (video visits, telephone visits, and routine telephone contact) were more common in the COVID cohort compared to the pre-COVID cohort (1% vs. 0%, 4% vs. 0%, and 25% vs. 14%, respectively).

**Table 4:** Characteristics of PrEP follow-up among persisters by cohort

	Total	Pre-COVID cohort	COVID cohort	Test statistic	P-value
	(N = 83)	(N = 51)	(N = 32)		
Bacterial STI testing with each prescription, n (%)				2.456^&^	0.117
Yes	69 (83)	45 (88)	24 (75)		
No	14 (17)	6 (12)	8 (25)		
HIV testing with each prescription, n (%)				2.456^&^	0.117
Yes	69 (83)	45 (88)	24 (75)		
No	14 (17)	6 (12)	8 (25)		
Serum creatinine within 6 months of each prescription, n (%)				0.602^&^	0.438
Yes	61 (74)	39 (77)	22 (69)		
No	22 (27)	12 (24)	10 (31)		
Visit type used, n (%)*				< 0.001^&&^	0.002
In-person visit	190 (79)	125 (85)	65 (68)		
Telephone visit	4 (2)	0 (0)	4 (4)		
Video virtual visit	1 (< 1)	0 (0)	1 (1)		
Routine telephone contact	45 (19)	21 (14)	24 (25)		
Missing	2 (< 1)	1 (< 1)	1 (1)		

Notes: * Visit level variable.

^&^ Chi-square value from chi-square test.

^&&^ Table probability value from Fisher exact test.

Results of the multivariable robust Poisson models are presented in Table 5. In the final model, we detected a significant interaction between cohort and the variables race/ethnicity and condom use during the follow-up period when predicting PrEP persistence. Among patients identifying as non-Hispanic White, the COVID cohort had 64% lower PrEP persistence than the pre-COVID cohort [aIR (95% CI), 0.36 (0.20, 0.64)]. In contrast, among patients who identified as a race/ethnicity other than non-Hispanic White, there was no significant difference in PrEP persistence between the COVID and pre-COVID cohorts [aIR (95% CI), 0.68 (0.41, 1.12)]. Condom use in the follow-up period was predictive of PrEP persistence in the COVID cohort [(aIR (95% CI), 3.71 (1.68, 8.22)] but was not predictive of PrEP persistence in the pre-COVID cohort [(aIR (95% CI), 1.37 (0.85, 2.22)]. PrEP persistence was associated with age such that a one-year increase in age was associated with an over 1% increase in the likelihood of PrEP persistence [aIR (95% CI), 1.01 (1.00, 1.02)]. Although not statistically significant, reporting more than ten sexual partners in the past year was associated with an 18% increase in PrEP persistence [aIR (95% CI), 1.15 (0.91, 1.52)].

**Table 5:** Predictors of PrEP persistence*^#^

	Final model Adjusted IR (95% CI)	P Value
Cohort		
Cohort effect among Non-Hispanic White		
COVID	0.36 (0.20, 0.64)	< 0.001
Pre-COVID	Ref	
Cohort effect among other race/ethnicity		
COVID	0.68 (0.41, 1.12)	0.131
Pre-COVID	Ref	
Cohort effect among condom used during the follow-up period		
COVID	0.81 (0.49, 1.33)	0.408
Pre-COVID	Ref	
Cohort effect among condom not used during the follow-up period		
COVID	0.30 (0.14, 0.66)	0.003
Pre-COVID	Ref	
Age	1.01 (1.00, 1.02)	0.007
Sex assigned at birth		
Female	0.78 (0.36, 1.70)	0.538
Male	Ref	
Race/Ethnicity		
Race/Ethnicity effect among COVID cohort		
Non-Hispanic White	0.65 (0.39, 1.06)	0.084
Other race/ethnicity	Ref	
Race/Ethnicity effect among pre-COVID cohort		
Non-Hispanic White	1.22 (0.87, 1.71)	0.248
Other race/ethnicity	Ref	
Condom use during the follow-up period		
Condom use during the follow-up period effect among COVID cohort		
Yes	3.71 (1.68, 8.22)	0.001
No	Ref	
Condom use during the follow-up period effect among pre-COVID cohort		
Yes	1.37 (0.85, 2.22)	0.200
No	Ref	
Neighborhood deprivation index	1.05 (0.92, 1.19)	0.455
Injection drug use		
Yes	1.18 (0.89, 1.58)	0.256
No	Ref	
Bacterial STI in the past year		
Yes	0.78 (0.46, 1.32)	0.351
No	Ref	
Number of sexual partners in the last year		
> 10	1.18 (0.91, 1.52)	0.214
<= 10	Ref	

Notes: * Models included complete cases only, i.e., the observation was deleted if any variable in the model had a missing value.

^#^ PrEP persistence was defined as any prescription for PrEP provided more than 60 days after the initial prescription.

## Discussion

In an urban sexual health clinic, patients who initiated PrEP in the four months prior to the COVID pandemic had significantly less persistence on PrEP than those who initiated PrEP one year prior. For those who persisted on PrEP, continuous PrEP coverage was more common in the pre-COVID cohort than in the COVID cohort. While this was likely multifactorial, based upon patient report, it was in part due to changed sexual behaviors and perceived risk of HIV acquisition during the COVID pandemic. This is consistent with previously published studies that have documented decreased PrEP use with changed sexual behaviors during the COVID pandemic [10, 22, 23]. Surveys of MSM on PrEP in the Southern United States and New England showed significant reductions in the number of sexual partners following the COVID-19 pandemic with the former showing a rebound in the number of sexual partners at the end of their study period (April – June 2020) and the latter showing progressively fewer sexual partners at the end of their study period (April-July 2020 compared to March-April 2020) [10, 24]. We do note that in both of our cohorts, almost a third of non-persisters were diagnosed with a bacterial STI at our clinic in the year after PrEP initiation, suggesting an ongoing risk of HIV acquisition despite ceasing PrEP.

While several other clinics shifted care almost entirely to telemedicine (tele-PrEP) [7–9, 12, 13], our clinic remained open for and continued to recommend in-person visits throughout the pandemic. The majority of visits for our patients on PrEP took place in-person during the COVID era, although the total visits decreased in volume. Formal telehealth visits were infrequently used, although we continued to employ routine telephone contact to support adherence. Our in-person care model changed in the pandemic with the elimination of walk-in services including walk-in testing. Despite this, the large majority of patients in our clinic continued to receive recommended bacterial STI and HIV screening. Some of this sustained screening was done through collaboration with community partners that continued to operate with flexibility and offer walk-in services during the pandemic. Adherence to recommended serum creatinine testing decreased during the pandemic, although recommended creatinine checks did continue for over two-thirds of patients and the change from pre-COVID testing was not statistically significant.

In our pre-COVID cohort, PrEP persistence was associated with multiple well-documented demographic features, with patients persisting on PrEP being older and more likely to identify as non-Hispanic White than other racial/ethnic groups [25]. In our COVID cohort, those factors were no longer predictive of persisting on PrEP. Although not statistically significant, in our COVID cohort, patients who identified as non-Hispanic Black, Hispanic/Latinx, or Other were more likely to persist on PrEP than patients identifying as non-Hispanic White. This trend is the opposite of that documented elsewhere, with many other clinics noting exacerbations of underlying disparities during COVID [1, 8, 26, 27]. One possible contributor to this trend of diminished racial/ethnic disparities in PrEP persistence is our clinic’s flexible care model, including the continued availability of in-person visits. As has been previously noted [27], reliance on telemedicine risks excluding those with less access to technology. For sexual health care in particular, the need for a private space in which to conduct visits may be a further barrier to equitable virtual care. A large community health center in our city serving a similar risk population (predominantly MSM) that adopted a near complete (98%) transition to telehealth early in the pandemic found that this model best preserved PrEP access for patients who are older, non-Hispanic, and White [8]. We also note that some patients may have a strong alliance with our clinic staff, many of whom are bilingual and have racial and ethnic identities that reflect the diversity of the patients we serve.

Our study has several strengths and limitations. We describe a care model for PrEP that continued to allow for in-person visits, which is not well described elsewhere. The population served by our clinic is diverse, with a high proportion of non-Hispanic Black and Hispanic/Latinx patients and approximately half of patients born outside the United States. We have detailed information on patient demographics and characteristics of persistence (short-term, interrupted, or continuous) and quality of care measures such as STI, HIV, and creatinine screening. Lastly, our study has a one-year follow-up, period whereas many publications to date describe the early months of the COVID pandemic and may no longer reflect characteristics of PrEP care. One limitation of our study is that for the majority of patients who discontinued PrEP, follow-up with our clinic was limited and thus we do not have a complete understanding of reasons for PrEP discontinuation. It is also possible that some patients continued on PrEP through other providers and granular details about their experience are therefore lacking in our data. Our definition of PrEP persistence was based upon patients seeking medication refills. We do not have access to data that verify if prescriptions were filled or taken with enough frequency to confer protection. For patients who reported transferring care for PrEP, we were not able to verify that additional prescriptions were sought or taken. These factors may have overestimated PrEP persistence in our clinic patients. Conversely, it is possible that some patients sought care outside of our medical system and did not inform our clinic of transferring care. In these instances, PrEP persistence would be underestimated. Our data are limited to the experience of one sexual health program in an urban setting and may not reflect the experiences of other clinics and patient populations. Our relatively small sample size limits our power to detect differences between subgroups and the lack of a contemporaneous comparator group limits our ability to infer causality. There is a narrow distribution of ages among our patients on PrEP, making the clinical significance of associations between age and PrEP persistence less clear.

In summary, our study found that rates of PrEP persistence declined during the COVID pandemic. However, in our clinic’s experience, underlying disparities were diminished rather than exacerbated during this time. We propose that flexible models of care, including continued opportunities for in-person care, may be one contributor to this trend. We do note that in both cohorts, many patients who did not persist on PrEP were diagnosed with a bacterial STI in our clinic in the following year, suggesting ongoing risk of HIV acquisition. As rates of STIs continue to rebound [28, 29], active efforts to reengage patients in PrEP care will be important.

## Data Availability

The data that support the findings of this study are not openly available due to sensitivity of the data, but may be available from the corresponding author upon reasonable request.
